# Psoriatic Arthritis Features and Risk of Cardiovascular Events: A Nationwide Cohort Study

**DOI:** 10.7759/cureus.97377

**Published:** 2025-11-20

**Authors:** Roel Sanchez Baez, Arthur Kavanaugh, Monica Guma, Joel M Kremer, George Reed

**Affiliations:** 1 Department of Medicine, University of California San Diego, La Jolla, USA; 2 Department of Medicine, Corrona Research Foundation, Delray Beach, USA; 3 Department of Medicine, University of Massachusetts, Worcester, USA

**Keywords:** cardiovascular risk, dactylitis, enthesitis, major adverse cardiovascular events (mace), psoriatic arthritis (psa)

## Abstract

Objective: This study aimed to assess the specific clinical features of psoriatic arthritis as predictors of cardiovascular (CV) risk.

Methods: Data were drawn from the CorEvitas observational US registry database of 5,131 patients with psoriatic arthritis. Out of these, 3,696 were included because they had no history of a CV event and had at least one follow-up visit. Participant characteristics were assessed at baseline visits, and participants were tracked until their last follow-up visit or the occurrence of a CV event. The mean tracked follow-up time was 3.69 years (SD ±2.87). Dactylitis and enthesitis were designated as the primary predictors of CV events. Secondary predictors were tender and swollen joint counts, nail involvement, and psoriasis body surface area percentage. Baseline characteristics were compared between those with dactylitis vs. those without and those with enthesitis vs. those without, using Fisher's exact test for categorical variables and the Kruskal-Wallis test for continuous variables. Unadjusted Kaplan-Meier curves were estimated. Cox regression models estimated CV risk (hazard ratios).

Results and conclusion: No statistically significant association between designated predictors and the development of CV events was observed. Dactylitis was more prevalent in the group with enthesitis than in the group without enthesitis (p<0.001). Enthesitis was also more prevalent in the group with dactylitis than in the group without dactylitis (p<0.001). Numbers of tender and swollen joints were significantly increased both with dactylitis and enthesitis (p<0.001 for all four analyses). Our results contrast with prior studies reporting associations of dactylitis with CV events and enthesitis with CV risk.

## Introduction

Patients with psoriatic arthritis (PsA) have an increased incidence of cardiovascular disease (CVD) compared to the age- and sex-matched general population [1]. In a study in which the leading cause of death was circulatory, the standardized mortality ratio was 1.59 for women and 1.65 for men in a sample of patients with PsA compared with the general population [2]. Patients with PsA have been reported to have a higher prevalence of cardiovascular (CV) risk factors, such as hyperlipidemia [3,4] and hypertension [3-5], and CV outcomes such as ischemic heart disease [3], atherosclerosis [3], peripheral vascular disease [3], congestive heart failure [3], cerebrovascular disease [3], and myocardial infarction (MI) [5]. Patients with PsA have been found to have higher rates of CV risk factors and CV events than those with skin-only psoriasis, suggesting inflammatory joint disease may contribute to increased CV risk in PsA patients [6].

The Framingham Risk Score (FRS) is a tool used to calculate CVD risk in the general population. The FRS uses age, sex, smoking status, total cholesterol, high-density lipoprotein (HDL) cholesterol, systolic blood pressure, and treatment for blood pressure so as to predict MI or death [7].

The FRS has been reported to underestimate CVD risk in patients with severe psoriatic plaques [8]. Some such patients are classified into the low- or intermediate-risk categories on the FRS, but are later found to be at higher risk when assessed by other modalities, such as the presence of atherosclerotic plaque on carotid ultrasound. (More than half of a sample of 100 severe psoriasis patients in this study were later reclassified [8].) Patients with PsA have been found to be more frequently reclassified into higher-risk categories than patients with psoriasis alone [9].

A recently presented abstract introduced a model that predicted CVD risk with higher sensitivity and specificity than the FRS in a population of patients with systemic lupus erythematosus (SLE) by combining SLE features with the FRS in a prediction model [10]. We hypothesize that considering PsA features may enhance CVD risk prediction beyond what is captured by FRS metrics.

We sought to assess the specific clinical features of PsA that might serve as predictors of CVD risk in order to identify candidates that could be used to build a modified FRS for patients with PsA.

## Materials and methods

Study setting

The US CorEvitas (formerly Corrona) PsA/spondyloarthritis (SpA) registry, a prospective, multicenter, observational, disease-based registry, was started in April 2013.

This study was conducted in accordance with the ethical principles of the Declaration of Helsinki. All participating investigators were required to obtain full board approval for conducting research involving human subjects. Sponsor approval and continuing review were obtained through the New England Institutional Review Board (IRB) (approval number: 13-073). For academic investigative sites that did not receive a waiver to use the central IRB, approval was obtained from the respective governing IRBs, and documentation of approval was submitted to the sponsor prior to initiating any study procedures. All registry subjects were required to provide written informed consent prior to participating.

Population

Psoriatic features were reported on provider forms. Dactylitis and enthesitis were designated as primary predictors. Tender and swollen joint counts, nail involvement, and psoriasis body surface area (BSA) percentage were designated as secondary predictors. Registry data as of December 31, 2023, were used.

Inclusion criteria included a diagnosis of PsA and having at least one follow-up visit. The enrollment form gave information from the provider on the history of CV events, and a prior history of CVD was exclusionary. A total of 3,696 patients in the CorEvitas database met the criteria and were included. 

Outcome

CV events were defined based on the Framingham definition [7] (see Table 1 for Framingham terms and comparative registry CV events captured). CV events were provider-reported. When a CV event was reported, an additional targeted adverse event (TAE) report was completed by the site for additional details to validate the event and avoid duplicate events. The TAE reports were derived from hospital records or those of other treating physicians. The pharmacovigilance group at CorEvitas compiled event data, eliminating duplicates and contacting sites if insufficient information was entered.

**Table 1:** Framingham term and comparative registry cerebrovascular events captured PsA: psoriatic arthritis

2008 Framingham Risk Score term [6]	PsA registry term
Coronary heart disease	Coronary artery disease; acute coronary syndrome; angina (stable and unstable); myocardial infarction; cardiac revascularization; cardiac arrest
Cerebrovascular	Transient ischemic attack; stroke
Heart failure	Systolic congestive heart failure; diastolic congestive heart failure
Peripheral artery disease	Peripheral artery disease; urgent peripheral arterial revascularization; peripheral ischemia/gangrene

Per the Framingham Heart study, "The risk score calculators are available for public use and do not require any specific permission or licensing fees" [11]. 

Exposure time

The index date was the patient registry enrollment visit, and participants were followed until their last follow-up visit or the occurrence of a first CV event, whichever occurred first.

Exposures

Participant characteristics assessed included age, sex, smoking status, body mass index (BMI), and psoriatic features. These clinical data were reported at the time of an observational study visit.

Statistical methods

Patient characteristics at the index date were compared by dactylitis and enthesitis status. Differences in characteristics were tested using a Fisher's exact test for categorical variables and the Kruskal-Wallis test for continuous variables. P-values are reported. The overall CV rate was estimated with a 95% confidence interval (CI). Unadjusted Kaplan-Meier curves were estimated to illustrate time to CV event by dactylitis and enthesitis. Unadjusted Kaplan-Meier curves were chosen over adjusted survival curves because they display the data nonparametrically and reveal potential violations of proportional hazards. In contrast, adjusted survival curves are semi-parametric and can be depicted in various ways depending on how confounding variables are specified. A test for violation of the proportional hazards assumption was carried out using the Schoenfeld residuals. (A significant result indicates a violation of the assumption.) Cox regression models estimated CV risk using hazard ratios (HR) with 95% CIs. P-values were presented, testing the null hypothesis of HR=1. For each predictor (primary and secondary), three models were estimated: unadjusted; adjusted for gender, age, and smoking status; and adjusted for gender, age, smoking status, BMI, duration of PsA, and race. Covariates were chosen a priori by clinical investigators because they were hypothesized to influence the relation between psoriatic features and CV risk. When all covariates were considered together, approximately 5% of observations had incomplete data.

P-values of <0.05 designated statistical significance. Since we had two primary outcomes, no adjustments for multiple comparisons were needed. When data were missing for a metric contributing to an analysis, individuals were excluded from that analysis. All analyses were carried out using Stata 18.0 (StataCorp LLC, College Station, TX, USA).

## Results

The CorEvitas database included 5,131 patients with PsA. Enrollment ranged from 4/1/2013 to 10/26/2023. Of these, 1,153 had no follow-up, and 282 had a prior history of a CV event. This left 3,696 with no history of a CV event and at least one follow-up visit. The total tracked follow-up time was 13,656 years. The mean tracked follow-up time per person was 3.69 years (SD ±2.87). Ninety-four CV events were reported, yielding an incidence rate of 6.9/1000 person-years (95% CI: 5.6-8.4). The most common CV events were revascularization (26%), MI (18%), coronary artery disease (15%), and stroke (13%).

Participant characteristics in those with and without dactylitis and those with and without enthesitis are represented in Table 2. Those without dactylitis were older than those with dactylitis. Approximately 2/3 of those with enthesitis were female, whereas the sexes were evenly represented in those without enthesitis. BMI and diabetes prevalence were comparable in those without and with dactylitis and in those with and without enthesitis (though there was a slight statistically significant increase in BMI in those with enthesitis, the distribution of BMI categories remained comparable). Proportions in each smoking status category are comparable across groups.

**Table 2:** Participant characteristics The data has been represented as mean (SD) for continuous variables and n (%) for categorical variables. P-values were generated from Fisher's exact test for categorical variables and the Kruskal-Wallis test for continuous variables. P-values of <0.05 were considered statistically significant. *Fisher's exact test does not provide a single test statistic: Fisher's exact test is performed by calculating the probability of the data that is observed if the null hypothesis (no association) is true, by using all possible 2×2 tables that hypothetically could have been observed, for the same row and column totals as those that are observed in the data (these are sometimes referred to as the marginal totals). BSA: body surface area; BMI: body mass index; PsA: psoriatic arthritis

Characteristic	Dactylitis (n=470)	No dactylitis (n=3,226)	Test statistic	P	Enthesitis (n=925)	No enthesitis (n=2,771)	Test statistic	P
	Mean (SD)	Mean (SD)			Mean (SD)	Mean (SD)		
Age	51.38 (0.64)	53.34 (0.23)	7.44	0.006	53.07 (0.41)	53.10 (0.25)	0.04	0.846
BMI	31.45 (0.34)	32.06 (0.13)	2.71	0.100	32.39 (0.25)	31.84 (0.14)	4.23	0.040
Duration of PsA symptoms (years)	7.59 (0.40)	10.59 (0.18)	60.39	<0.001	10.59 (0.37)	10.08 (0.19)	0.03	0.867
Duration of PsA diagnosis (years)	4.64 (0.32)	6.97 (0.15)	71.83	<0.001	5.44 (0.26)	7.09 (0.16)	55.77	<0.001
Psoriasis BSA	5.32 (0.47)	5.44 (0.20)	4.07	0.048	5.23 (0.35)	5.49 (0.22)	0.87	0.352
Tender joint count (TJC68)	9.55 (0.52)	5.11 (0.15)	158.30	<0.001	12.09 (0.41)	3.50 (0.12)	602.63	<0.001
Swollen joint count (SJC66)	5.95 (0.34)	1.90 (0.06)	323.91	<0.001	3.58 (0.19)	2.03 (0.07)	69.49	<0.001
	N (%)	N (%)			N (%)	N (%)		
Sex								
Male	211 (45.1)	1,382 (43.0)	*	0.424	306 (33.2)	1,287 (46.7)	*	<0.001
Female	257 (54.9)	1,830 (57.0)			617 (66.8)	1,470 (53.3)		
Smoking status								
Never	277 (59.4)	1,806 (56.5)	*	0.141	515 (56.2)	1,568 (57.2)	*	0.054
Former	150 (32.2)	1,026 (32.1)			282 (30.8)	894 (32.6)		
Current	39 (8.4)	362 (11.3)			120 (13.1)	281 (10.2)		
BMI category								
Underweight (<18.5)	1 (0.2)	29 (0.9)	*	0.200	10 (1.1)	20 (0.7)	*	0.177
Normal weight (18.5 to <25)	79 (17.0)	479 (15.1)			136 (14.9)	422 (15.5)		
Overweight (25 to <30)	143 (30.7)	904 (28.5)			243 (26.6)	804 (29.6)		
Obese (≥30)	243 (52.1)	1,755 (55.4)			526 (57.5)	1,472 (54.2)		
Diabetes	59 (12.6)	413 (12.8)	*	0.941	118 (12.8)	354 (12.8)	*	1.000
BSA category								
High (≥4%)	153 (33.1)	960 (30.8)	*	0.332	279 (30.5)	834 (31.3)	*	0.679
Low (0-3%)	309 (66.9)	2,157 (69.2)			635 (69.5)	1,831 (68.7)		
Enthesitis	167 (35.5)	758 (23.5)	*	<0.001				
Dactylitis					167 (18.1)	303 (10.9)	*	<0.001

Most psoriatic features were more prevalent in the dactylitis and enthesitis groups than they were in the no dactylitis and no enthesitis groups, respectively. Compared to those without dactylitis, those with dactylitis had higher tender and swollen joint counts, a longer duration of PsA symptoms and diagnosis, and a higher prevalence of enthesitis. Increases in tender and swollen joints were observed in the group with enthesitis compared to the group without enthesitis. There was a statistically significant increase in dactylitis in the group with enthesitis vs. those without. Similarly, there was a statistically significant increase in enthesitis in the group with dactylitis vs. those without.

**Figure 1:**
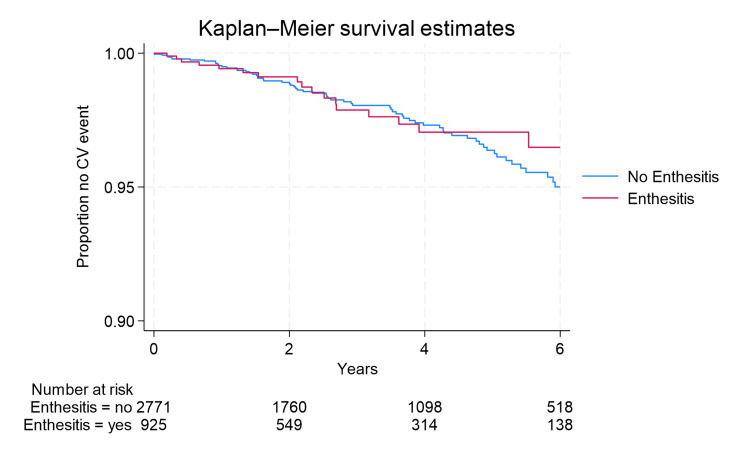
Kaplan-Meier survival curve for enthesitis CV: cardiovascular

**Figure 2:**
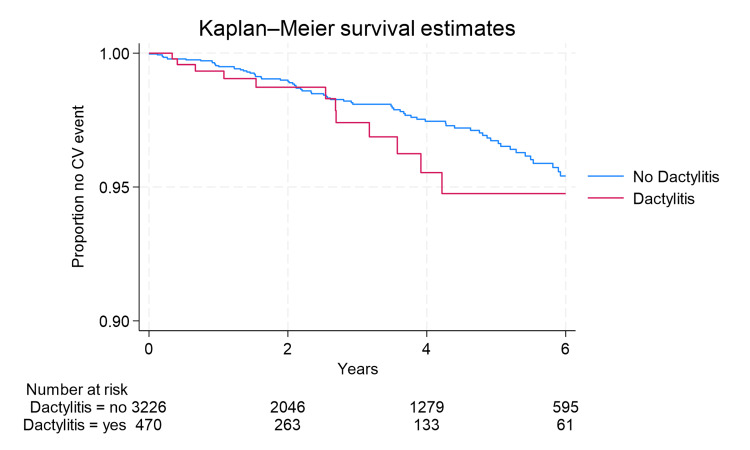
Kaplan-Meier survival curve for dactylitis CV: cardiovascular

Figure 1 and Figure 2 show estimated Kaplan-Meier curves comparing those with and without dactylitis and those with and without enthesitis. The curves do not present striking differences in the comparison groups as quantitated by the estimated HR. The test for violation of the proportional hazards assumption was not significant, indicating that the assumption should not be rejected.

Table 3 shows the estimated HRs with 95% CIs for each predictor and each model. Neither dactylitis nor enthesitis was a significant predictor of CV events in the unadjusted or adjusted models. Nor did any secondary predictors significantly relate to CV risk. HRs for dactylitis (HR=1.51) and swollen joints (HR=1.75 in the highest tertile) were greater than 1 in the adjusted model 2, but neither was statistically significant. Enthesitis and psoriasis BSA did not show an increased risk in this model.

**Table 3:** Estimated HRs with 95% CIs for each predictor and each model *Adjusted for gender, age, and smoking status. **Adjusted for gender, age, smoking status, BMI, PsA duration, and race. z=z-score; X(n)=chi-squared statistic with n degrees of freedom. The data has been represented as HR (95% CI). P-values are provided for Cox regression models. P-values of <0.05 were considered statistically significant. For the adjusted model 1, there were <1% data missing. For the adjusted model 2, there were an additional 5% data missing. The adjusted model 1 was run excluding cases with missing data in model 2, and the estimated HR varied only from the second decimal place onward, so changes in model 2 were due to adjustment for confounding not the elimination of the small subset with missing data. BMI: body mass index; PsA: psoriatic arthritis; CV: cardiovascular; HR: hazard ratios; BSA: body surface area

	Unadjusted model	Adjusted model 1*	Adjusted model 2**
	HR (95% CI)	HR (95% CI)	HR (95% CI)
Dactylitis	1.23 (0.67-2.26)	1.44 (0.78-2.66)	1.51 (0.81-2.79)
	p=0.498; z=0.50	p=0.240; z=1.14	p=0.194; z=1.30
Enthesitis	0.82 (0.49-1.38)	0.91 (0.53-1.57)	0.92 (0.53-1.61)
	p=0.461; z=-0.74	p=0.742; z=-0.33	p=0.263; z=-0.28
Tender joint quartiles			
0	Ref	Ref	Ref
1-2	1.30 (0.74-2.29)	1.40 (0.79-2.48)	1.45 (0.80-2.64)
3-7	0.81 (0.42-1.56)	0.90 (0.46-1.75)	0.97 (0.49-1.90)
>7	1.55 (0.94-2.58)	1.68 (0.98-2.87)	1.72 (0.98-3.01)
	p=0.191; χ(3)=4.75	p=0.174; χ(3)=4.98	p=0.188; χ(3)=4.79
Swollen joint tertiles			
0	Ref	Ref	Ref
1-2	1.46 (0.87-2.46)	1.50 (0.88-2.56)	1.62 (0.93-2.81)
≥3	1.70 (1.05-2.72)	1.58 (0.97-2.58)	1.75 (1.06-2.91)
	p=0.075; χ(2)=5.98	p=0.127; χ(2)=4.13	p=0.061; χ(2)=5.60
Nail involvement	1.28 (0.83-1.99)	1.28 (0.81-2.03)	1.40 (0.87-2.24)
	p=0.267; z=1.11	p=0.284; z=1.07	p=0.163; z=1.39
Psoriasis BSA			
0-3%	Ref	Ref	Ref
≥4%	0.93 (0.59-1.49)	1.04 (0.65-1.68)	1.09 (0.67-1.78)
	p=0.773; z=-0.28	p=0.862; z=0.17	p=0.720; z=0.36

## Discussion

Consistent with reports showing an association between dactylitis and enthesitis [12], dactylitis was significantly more prevalent in the group with enthesitis than in the group without enthesitis, and enthesitis was also more prevalent in the group with dactylitis than in the group without dactylitis. Also, the numbers of tender and swollen joints were significantly increased both with dactylitis and with enthesitis, as shown elsewhere [13,14]. Of note, we observed no statistically significant association between disease activity in different domains (dactylitis, enthesitis, tender joint count, swollen joint count, psoriasis BSA) and the development of CV events.

These findings differ from some reports in the literature. Specific features of PsA have been associated with both higher assessed CV risk and risk of major adverse cardiovascular events (MACE). An observational cohort analysis by Eder et al. (N=1,091) found that the number of dactylitic digits (i.e., dactylitis) independently predicted major CV events in patients with PsA [15]. Like ours, Eder et al.'s study followed patients over time to see if they developed CV events, but we used the binary presence or absence of dactylitis as a predictor, while Eder et al. used the number of dactylitic digits. Their outcome was restricted to MACE, while ours encompassed the broader Framingham definition of CV events. A study that scored CV risk by Systematic Coronary Risk Evaluation risk charts found an association between enthesitis and joint erosions and increased CV risk (N=140) [16]. The design of this study was cross-sectional and assessed CV risk, not the actual presence or absence of MACE. PsA patients with nail involvement have been found to have higher rates of carotid plaque than patients without PsA [17], and carotid plaque was a risk factor for CV events in PsA patients in a cohort analysis with a much smaller sample size than our own [18]. It is unclear what accounts for the differences between these other findings and our own, but differences in methodology may have played a role. Our sample size was also larger than any of the studies cited, which is a strength of the present study.

We were unable to identify candidate predictors that could be incorporated into a modified FRS for use in the setting of PsA. Nevertheless, the discordant findings between our findings and those of previous studies suggest that further investigations of this possible relationship are appropriate.

Our findings emphasize the importance of considering biases when evaluating studies. For example, one retrospective study that used the Inpatient Registry and Psoriasis Association cohort found an increased risk for CV mortality in participants who were admitted for psoriasis at a dermatological ward but not in outpatients (n=8,991 inpatients and 19,757 outpatients) [19]. In this case, referral bias could account for the significance of the relation observed. Referral bias is not an issue in the present study, where data for the entire cohort was collected in an outpatient setting.

Selection bias can be present in datasets that are not as stringent in eliminating patients who have had a MACE prior to enrollment. For example, a retrospective cohort study that used the UK General Practice Research database (n=130,976) included patients with prior MI [20]. HR calculation in this study was adjusted for participants with prior MI. In a follow-up cohort study using the same database (n=36,702), more stringent criteria were used, and participants with prior MI or stroke were excluded. Brauchli et al. found no increased risk for MI overall, only in a subset of participants with severe plaque psoriasis who were less than 60 years of age [21]. Our study did eliminate those with prior MACE.

Limitations of our study include the following: The study design is observational, precluding conclusions about causality. Confounding bias could have played a role in the finding of no statistical association between psoriatic features and CV events. Residual confounding by factors not included in our models, such as prior CVD, concomitant medication use (e.g., biologics, statins), or other comorbid conditions like diabetes or hypertension, could have masked a true association between psoriatic features and CV events. We did not measure whether participants received treatment, so we could not adjust for this. Missing data may have introduced bias. Some incidents may have been inconsistently recorded in patient records. The absence of model validation in independent cohorts limits the generalizability of the results to other populations.

Our mean follow-up period of 3.69 years should be adequate to capture CV risk, although longer duration investigations would provide further insights. A meta-analysis found that among 112 cohort studies using Cox regression analysis to assess CV outcomes, the median follow-up period was 7.3 years, with an interquartile range of 5.9-11.8 years [22]. 

## Conclusions

We observed no statistically significant association between assessed psoriatic features and the development of CV events, differing from reports in the literature which found a statistically significant association between dactylitis and MACE and enthesitis and assessed CV risk.

Further studies examining the prediction of CV events by psoriatic features are warranted using nationwide cohorts that are large enough to account for more stringent inclusion/exclusion criteria. Further studies are needed to indicate whether the continued suppression of PsA disease activity reduces CV risk regardless of the treatment used.

Our study has substantial strengths. We investigated CV events in a very large national PsA registry followed by mandated, detailed, and uniform clinical metrics that included enthesitis, dactylitis, tender and swollen joints, as well as prior comorbidities and CV status.
